# Utilization of functional agro-waste residues for oyster mushroom production: Nutritions and active ingredients in healthcare

**DOI:** 10.3389/fpls.2022.1085022

**Published:** 2023-01-04

**Authors:** Xu Zeng, Jiaxue Li, Xinkai Lyu, Tongyao Chen, Juan Chen, Xiaomei Chen, Shunxing Guo

**Affiliations:** Institute of Medicinal Plant Development, Chinese Academy of Medical Sciences and Peking Union Medical College, Beijing, China

**Keywords:** recycling of agro-waste, substrate formulation, edible fungi, nutritional composition, functional ingredients

## Abstract

A large amount of agro-industrial residues are produced from the planting, production and processing of traditional Chinese herbs. As a tonic, edible, and economical herb, *Codonopsis pilosula* root has been extensively developed into medicine and functional food. However, thousands of tons of aerial parts (stems, leaves, flowers and fruits) have been directly discarded after harvest each year. To utilise agro-wastes, *Pleurotus ostreatus* was cultivated on a basal substrate supplemented with *C. pilosula* stems and leaves (CSL). Physicochemical analyses revealed that the basal substrate mixed with CSL was more abundant in cellulose, hemicellulose, and most of micronutrients such as K, Ca, Mg, S, Fe, Zn and Mo. After the first flush, the fruit bodies in CSL group exhibited a higher fresh weight, a wider average pileus diameter and a lower moisture level. Nutrition analyses presented a higher protein content and a lower fat content in mushrooms from CSL group compared with control group. Interestingly, 14 amino acids (glutamine, arginine, valine, leucine, and etc.) and 3 micronutrients (Se, Fe and Zn) were increased after CSL addition to the substrate. Based on untargeted metabolomics, a total of 710 metabolites were annotated. Compared with control group, there were 142 and 117 metabolites significantly increased and decreased in the CSL group. Most of them were grouped into classes of amino acids and peptids, fatty acids, carbohydrates, terpenoids, and etc. Moreover, an abundance of phytometabolites from *Codonopsis* were detected in *P. ostreatus* from CSL group, including polyacetylenes or polyenes, flavonoids, alkaloids, terpenoids, organic acids, and etc. UPLC-MS/MS results demonstrated that lobetyolin content in the CSL group samples was 0.0058%. In summary, the aerial parts of *C. pilosula* processed for use in the production of edible mushroom is an emerging strategy to converting agricultural waste into functional foods.

## 1 Introduction

In East, Southeast, and central Asia, *Codonopsis pilosula* (Franch.) Nannf., a perennial herbaceous herb, is used for tonic production, edible herb and medicinal materials, and has been practiced on a considerable scale for hundreds of years (Gao et al., 2018; Luan et al., 2021). Radix Codonopsis (dried roots, ‘Dangshen’ in Chinese and ‘Tojin’ in Japanese) had a total yield of about 50,000 tons approximately, while thousands of tons of agro-wastes residues are produced after harvest each year. Most of the agro-residues, the withered stems and leaves of *C. pilosula* (CSL), were always discarded or incinerated directly (Zeng et al., 2022a). Resource waste, serious environmental pollution and enormous economic loss are the main problems.

Agro-industrial residues were widely investigated to generate value as food or health products. Recent studies have showed that agro-wastes from herbs contained a variety of functional substances (Madureira et al., 2020; Reguengo et al., 2022). Agro-waste residues of *C. pilosula* are also available and highly valuable resource. Previous studies reported that functional ingredients from the agro-waste residues of *C. pilosula* were similar to the constituents of the roots, including a variety of polyene and polyacetylene glycosides, polysaccharides, flavonoids, lignans and penoids (Chen et al., 2020). Approximately 230 phytometabolites have been isolated and identified from Radix Codonopsis, and 165 of them were detected in the *C. pilosula* stems and leaves (He et al., 2015; Zeng et al., 2022a). Moreover, some studies reported that *C. pilosula* stems and leaves were used as feed additives in the poultry and livestock industry to increase growth performance, strengthen immune system, improve antioxidative status, and regulate the intestinal microflora (Hong et al., 2019; Zeng et al., 2019). Fresh leaves and flowers of *C. pilosula* have also been explored as tea products. As we known, the agro-waste residues of *C. pilosula* are mainly lignocellulose materials. Edible fungi are common primary decomposers of woody residues. Thus, our study was designed to evaluate the potential application of *C. pilosula* stems and leaves in the edible fungus industry.

*Pleurotus* spp., commonly known as the oyster mushroom, is a kind of nutrient-rich food, which also presents a health care value to humans. The species of the genus *Pleurotus* occupy the third position in the production of edible mushrooms (Fernandes et al., 2015). Based on the data from China edible fungi association, the total yield of oyster mushrooms in 2019 reached 300,000 tons (Zeng et al., 2022b). Compared to other edible fungi, oyster mushrooms have shown several advantages in cultivation, including short growth cycle, few environmental controls, disease and pest resistance, and low producing cost (Wan-Mahari et al., 2020). Oyster mushrooms are highly nutritious food and provide a good source of protein, carbohydrates, vitamins, and micronutrients. In recent decades, fungal polysaccharides in *Pleurotus* spp. were widely reported to improve immunity, regulate gut microbiota, and exhibit antioxidation, antitumor and cholesterol-lowering activities (Jayakumar et al., 2011; Sharma et al., 2021). The metabolites in *P. pulmonarius* and *P. ostreatus* exhibited profound antileukemic potential and immunomodulatory effects (Ebrahimi et al., 2018). A recent pharmacological study also indicated that dietary fibers in *P. sajor-caju* could promote the abundance of small-chain fatty acids producing bacterial genera and inhibit the growth of intestinal pathogens (Maheshwari et al., 2021). Therefore, oyster mushrooms are important natural products for nutrient source and therapeutic diet.

In recent years, different agro-industrial residues have broad development prospects as substrates in mushroom production. The cultivation of *Pleurotus* spp. have been extensively studied in different steps, in particular the substrates from agro-industrial waste. In previous studies, coffee husks, blank and printed paper scraps, rice straw and banana straw have been supplemented into the formulation of the substrates ( (Ramos et al., 2011; Naozuka et al., 2012; Fernandes et al., 2015). Here, the cultivation of *Pleurotus ostreatus* with the substrates added *C. pilosula* stems and leaves (CSL, agro-waste residues) could promote the mycelial growth, shorten the producing period and increased mushroom yield. Moreover, nutritions and active ingredients in mushrooms cultured with CSL were investigated by chemometrics and metabolomics. Our study showed an emerging strategy to turning agricultural waste materials into functional foods.

## 2 Materials and methods

### 2.1 Samples

**Figure 1:**
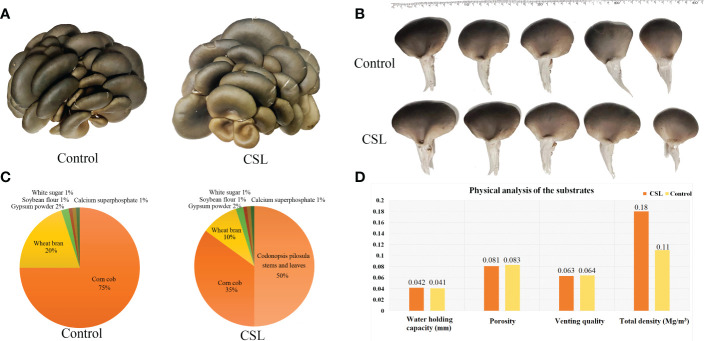
The fruiting body of *P. ostreatus* and physical parameter of the substrates. samples **(A)**, pileus diameter **(B)**, the formula of cultivation substrate **(C)**, physical analyses of substrates **(D)**.

A commercial strain of *P. ostreatus* (ITS sequencing, Genbank accession OL905951, **Figures 1A, B**) was obtained from Zehai Edible Fungus Specialized Cooperative (Shandong province, China) and cultivated on the basal substrate.

Agro-waste residues were collected from in the major production areas of Radix Codonopsis in Changzhi, Shanxi province (Zhendong Pharmaceutical Co., Ltd.). After washing, *C. pilosula* stems and leaves (CSL) were dried and crushed to a coarse powder. The formula of cultivation substrate was shown in **Figure 1C**. The basal substrate is comprised of corn cob, wheat bran, gypsum powder, soybean flour, white sugar, and calcium superphosphate. The groups were designated the CSL and control groups. In the control group, 500 g of basal substrates were prepared, with the moisture content of 65% and the pH value of 6.5. In the CSL group, 250 g of CSL coarse powder and 250 g of basal substrate were used as cultivation substrate, with the moisture content of 65% and the pH value of 6.5. Then, 500 g of each substrates were placed in HDPE (high-density polyethylene) bags and autoclaved at approximately 120°C for 3 h. Moreover, physicochemical analyses of two substrates were performed by a qualified third institutions (Nanjing Convinced-Test Technology Co., Ltd). Next, each bag was inoculated with ~3 g of *P. ostreatus* grown on basal substrate and kept at 25°C in the dark to encourage mycelial growth within 25 days. Later, a hole (~3 cm diameter, one per bag) was made in the side of the bag. Each bag was placed at 10-15°C and 80-85% humidity, until mushroom formation. After the first fruiting, the fruiting bodies from control group and CSL group were collected, immediately chilled in liquid nitrogen and stored at -80°C.

### 2.2 Nutrient contents

The nutrient contents of mushrooms (six replicates of CSL and control groups) were detected according to the National Standards of the People’s Republic of China (NSPRC). The fat was determined by the acid hydrolysis method (NSPRC GB 5009.6-2016). The protein was estimated determined by Kjeldahl method (NSPRC GB 5009.5-2016). The total saccharide content was analyzed by the phenol-sulfuric acid method (NSPRC GB/T 15627-2009). The amino acid component was detected by an automatic amino acid analyzer (NSPRC GB/T 15627-2009). Se, Fe, Mn, and Zn were investigated by inductively coupled plasma mass spectrometry (ICP-MS, NSPRC 5009.268-2016) and inductively coupled plasma optical emission spectrometry (ICP-OES, NSPRC 5009.13-2017).

### 2.3 Metabolomics analysis

Referring to the method recorded in previous studies (Ma et al., 2021; Zeng et al., 2022b), six biological replicates of mushrooms from CSL and control group were assayed for sample extraction and metabolomics analysis. In brief, 50 mg of each sample was extracted with a methanol buffer with internal standard (0.4 mL 80% aqueous, and 20 μL 0.3 mg/mL 2-chloro-D-phenylalanine) for 30 min in an ultrasonic bath. The extraction was incubated for 30 min at -20°C, followed by centrifugation at 13,000 × g at 4°C for 15 min. After filtering by a microporous membrane (0.22-μm pore size), 200 μL of supernatant was used for UPLC-MS analysis. To monitor the stability and repeatability, 10 μL of supernatant from each test sample was pooled into a quality control (QC) sample and injected every six test samples throughout the analytical run.

Untargeted metabolomics analysis was performed on a UPLC system (Waters, Milford, USA) equipped with a Q Exactive HF-X Mass Spectrometer (Thermo Fisher Scientific, San Jose, CA). Acquity BEH C18 column (100 mm × 2.1 mm i.d., 1.7 μm; Waters, Milford, USA) was used for LC separation. The column temperature and injection volume were 40°C and 2.00 μL. The solvent consisted of water (phase A) and acetonitrile (phase B) with 0.1% (v/v) acetic acid respectively.

The following gradient was used for separation with flow rate at 0.40 mL/min: 5-20% B over 0-3 min, 20-95% B over 3-9 min, and 95% B over 9-13 min, 5% B over 13-16 min. Based on mass spectrometer equipped with an electrospray ionisation (ESI) source, MS-acquisition was performed in both positive and negative ion mode. All other parameters were as follows: spray voltage (+) and (−), 3500 and 2800 V; sheath gas, 40 arb; capillary temperature, 320°C; auxiliary gas, 10 arb; probe heater temperature, 400°C; normalised collision energy (NCE), 20-40-60 V; and mass spectrum scanning range, 70-1050 m/z.

The raw data were converted using Progenesis QI 2.3 (Waters Corporation, Milford, USA) for baseline filtration, peak identification, integration, retention time correction, peak alignment, and uniformisation. Subsequently those preprocessing data generated a matrix containing the retention time (RT), mass-to-charge ratio (m/z), and peak intensity. The data matrix was converted using SIMCA-P+ 14.0 to perform principal component analysis (PCA) and orthogonal partial least squares discriminate analysis (OPLS-DA) analyses. Differential metabolites were identified based on the combination of variable influence on projection (VIP) > 1, p < 0.05, and |Log2(fold_change)| > 1 in OPLS-DA analysis. Metabolites were annotated first using the experimental spectral data (m/z tolerance <10 ppm) and analysed for their biological functions from public databases: METLIN (https://metlin.scripps.edu/), Human Metabolome (http://www.hmdb.ca/), and KEGG databases (using MetaboAnalyst, https://www.metaboanalyst.ca/). For phytochemical compounds, more than 230 metabolites identified in *C. pilosula* from previous studies were also analysed separately in our data.

### 2.4 Determination of lobetyolin by LC-MS/MS

Lobetyolin is a standard composition to evaluate the quality of Radix Codonopsis. According to the Chinese Pharmacopoeia (2020) and the previous studies (Dong et al., 2021; Zeng et al., 2022b), lobetyolin in oyster mushrooms from CSL and control group was extracted by the ultrasound-assisted method. In brief, 10 g of each dried samples was precisely weighed and introduced into a conical flask with 50 mL of methanol. The flask was sealed and sonicated in a water bath operating at 40 kHz, 100 W and 30 min. After 10 minutes, the supernatant fluid was collected, diluted 100-fold, filtered through a 0.22-µm microporous membrane, and stored at 4°C until UPLC-MS/MS analysis.

The standard substance of lobetyolin was accurately weighed and dissolved in methanol to a concentration of 1 mg/mL. After dilution with methanol, the standard solutions with final concentrations of 0.5, 1, 5, 10, 50, 100, and 200 ng/mL were prepared.

Quantitative analysis was carried out using an Agilent 1290-6460 LC-MS/MS instrument (Agilent, USA), and an Agilent C18 column (2.1 mm × 100 mm, 3 μm) with column temperature at 35°C. Mobile phase was water containing 0.1% formic acid (A) and methanol (B) with with gradient conditions as follows: 0 min, 40% B; 0–1 min, 40–60% B; 1–3 min, 60–90% B; 3–4 min, 90% B; and 4.01–6 min, 40% B. The flow rate was 0.3 mL/min. The injection volume was 5 μL. The lobetyolin was achieved in negative ESI mode with dynamic Multiple Reaction Monitoring (MRM). The optimized conditions were listed as follows: ion spray voltage, 5500 V; collision energy, 30 V; and capillary temperature, 550°C. Lobetyolin produced a precursor ion of m/z 441.4, corresponding to the [M+COOH]-. Likewise, the mass transition patterns m/z 441.4 + 179.0 and 441.4 + 185.4 were selected for the identification and quantification. Data were analyzed with SPSS 19.0. Student’s t-tests were used and P-values ≤ 0.05 were considered significant.

## 3 Results and discussion

### 3.1 Physicochemical analysis of the substrates

**Figure 2:**
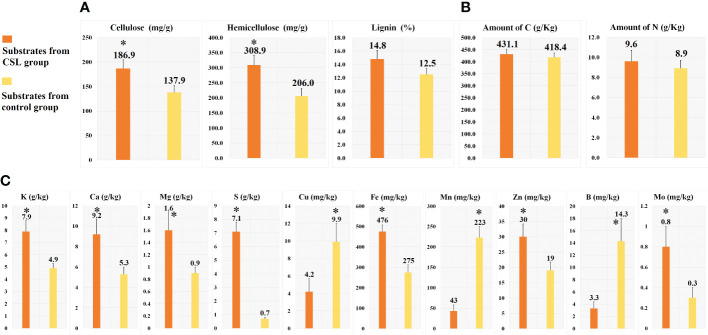
Chemical analyses of the substrates. cellulose, hemicellulose and lignin **(A)**, macronutrients **(B)** and micronutrients **(C)**. *, Statistically significant p < 0.05.

In comparing substrates from CSL and control group (**Figures 1D** and **2**), physicochemical analyses indicated that the basal substrate mixed with agro-waste residues was more abundant in cellulose (186.9 vs 137.9 mg/g), hemicellulose (308.9 vs 206.0 mg/g), and most of micronutrients K, Ca, Mg, S, Fe, Zn and Mo. However, the contents of Cu, Mn and B were decreased in the substrates of CSL group compared with control. In addition, there was no different in the amount of lignin, macronutrients C and N, and most of physical parameter, such as water holding capacity, porosity, venting quality and total density between the two substrates.

### 3.2 Nutrients in oyster mushroom samples

After the first flush, the fruit bodies obtained in the CSL group resulted in a higher fresh weight (90.23 ± 11.52 vs. 59.17 ± 7.22 g), a wider average pileus diameter (7.71 ± 0.79 vs. 6.96 ± 0.81 cm) and a lower moisture level (81.82 ± 1.25 vs. 86.24 ± 2.77 g/100 g) as compared to that of control group (**Table 1**). Previous studies have shown that oyster mushrooms grown on a variety of substrates present different levels of moisture, such as 88.06 and 85.64 g/100g for *P. ostreatus* cultivated in banana and rice straw, 83.17 and 88.08 g/100g for *P. sajor-caju* cultivated in banana and rice straw (Bonatti et al., 2004), 89.17 and 89.00 g/100 g for *P. ostreatus* and *P. eryngii* obtained in local supermarkets (Reis et al., 2012); and 90.3 and 91.0 g/100 g for *P. ostreatus* cultivated in blank and printed paper scraps (Fernandes et al., 2015).

**Table 1:** Nutritional value, amino acid content and elemental concentrations of *P. ostreatus* (mean ± SD).

	Control	CSL group
Nutritional value		
Fresh weight (g)	59.17 ± 7.22	90.23 ± 11.52*
Pileus (cm)	6.96 ± 0.81	7.71 ± 0.79*
Moisture (g/100 g)	86.24 ± 2.77	81.82 ± 1.25*
Protein (g/100 g)	16.58 ± 1.26	26.67 ± 3.08*
Fat (g/100 g)	3.33 ± 0.27	2.45 ± 0.08*
Total saccharide (%)	50.36 ± 3.13	50.37 ± 4.41
Amino acid (g/100 g)		
Aspartic acid	1.17 ± 0.05	1.80 ± 0.02*
Threonine	0.58 ± 0.02	0.80 ± 0.01*
Serine	0.54 ± 0.02	0.74 ± 0.05*
Glutamine	1.99 ± 0.14	2.56 ± 0.08*
Proline	0.52 ± 0.02	0.71 ± 0.01*
Glycine	0.59 ± 0.03	0.75 ± 0.02*
Alanine	0.82 ± 0.05	1.03 ± 0.01*
Valine	0.60 ± 0.04	0.70 ± 0.01*
Methionine	0.16 ± 0.01	0.17 ± 0.01
Isoleucine	0.49 ± 0.04	0.65 ± 0.01*
Leucine	0.96 ± 0.03	1.22 ± 0.06*
Tyrosine	0.47 ± 0.02	0.53 ± 0.04
Phenylalanine	0.58 ± 0.02	0.75 ± 0.03*
Lysine	0.79 ± 0.04	1.09 ± 0.02*
Histidine	0.27 ± 0.02	0.37 ± 0.01*
Arginine	0.63 ± 0.03	0.92 ± 0.02*
Total amino acid	11.13 ± 0.49	14.96 ± 0.07*
Mineral (mg/kg)		
Se	0.2033 ± 0.0071	0.2430 ± 0.0014*
Fe	47.86 ± 0.91	59.53 ± 4.50*
Mn	9.24 ± 037	7.71 ± 0.11*
Zn	71.88 ± 3.22	79.34 ± 2.88*

The results are expressed on a dry weight basis from the first flush except for the fresh weight, amino acid content and moisture (* = statistically significant, p < 0.05).

Nutrient composition was different between the two groups. Compared with control group, there was a higher protein content (26.67 ± 3.08 vs 16.58 ± 1.26 g/100g), a lower fat content (2.45 ± 0.08 vs 3.33 ± 0.27 g/100g), and a similar carbohydrate content were observed in the CSL group (shown in **Table 1**). In our study, the protein contents of oyster mushroom was similar with or higher than the values reported by previous studies: 19.9-34.7 g/100 g for *P. ostreatus* cultivated in wheat straw supplemented with sugar beet (Manzi et al., 1999), 21.0 g/100 g for *P. ostreatus* cultivated in wheat straw (Patil et al., 2010), 9.29 and 9.62 g/100g for oyster mushrooms cultivated on paper scraps and coffee husks, respectively (Fan et al., 2000; Aguilar-Rivera et al., 2012). Moreover, the total fat content of the oyster mushroom in our study (2.45 and 3.33 g/100g) was lower than the values obtained from samples cultivated on banana straw and rice straw (5.97 and 6.32 g/100 g), respectively (Bonatti et al., 2004); although higher than the values obtained from *P. ostreatus* grown on blank and printed paper scraps (1.18 and 1.68 g/100 g), respectively (Fernandes et al., 2015).

Subsequently, total amino acids contents of CSL group were obviously higher than those of control group (14.96 ± 0.07 vs. 11.13 ± 0.49 g/100 g). In terms of individual amino acids, 14 amino acids were more abundant in samples from CSL group than control group. Of these, the comparison between the two groups shows that the oyster mushroom in the CSL group contained more aspartic acid, threonine, serine, glutamine, glycine, alanine, valine, leucine, isoleucine, histidine, and arginine.

Meanwhile, the Se (0.2430 vs 0.2033 mg/kg), Fe (59.53 vs 47.86 mg/kg) and Zn (79.34 vs 71.88 mg/kg) concentrations in the CSL group were higher than those in the control group. However, the Mn (7.71 vs 9.24 mg/kg) concentrations were decreased after CSL addition to the substrate. Interestingly, similar results were investigated in the micronutrient analyses of the substrates (shown in **Figure 2**). Here, the concentrations of Se, Mn, and Zn (**Table 1**) from our results were similar to the previous reports (Kalac, 2009; Naozuka et al., 2012). For example, *P. ostreatus* were cultivated with coffee husks containing 0.12 mg/kg of Se, 70 mg/kg of Zn, and 10mg/kg of Mn. In addition, the concentrations of Fe (47.86 vs 59.53 mg/kg from control and CSL group) were lower than the data described in the previous reports (Koutrotsios et al., 2020). Their results were 80-130 mg/kg in *P. ostreatus* cultured on seven different substrates. However, according to the standard suggested by World Health Organization, the maximum limit of the Fe is 15 mg/kg. In the previous study (Karami et al., 2021), the Fe concentrations of many edible mushrooms (ranged from 97.2-3919 mg/kg) were higher than the WHO allowed limit, due to the ability of the mushroom to absorb Fe. Moreover, the recommended dietary intake (RDI) of Fe is 10-50 mg/day (Karami et al., 2021).

In summary, our results implied that the CSL addition to the substrate could contribute significantly to the overall nutritional value, amino acid content, and elemental concentration of oyster mushrooms.

### 3.3 Overview of metabolomics

Untargeted metabolomics of samples from the CSL group and the control (each group with six biological replicates) were carried out using the protocol with UPLC-MS and an integrated informatics pipeline. After removal of noise, redundancy, and poor quality signals, 6568 and 8584 spectral signals were collected by positive and negative ion modes, respectively. Subsequently, accurate m/z values were used for metabolite annotation. In total, 710 metabolites were annotated by our integrated bioinformatics pipeline (**Supplementary Table S1**).

**Figure 3:**
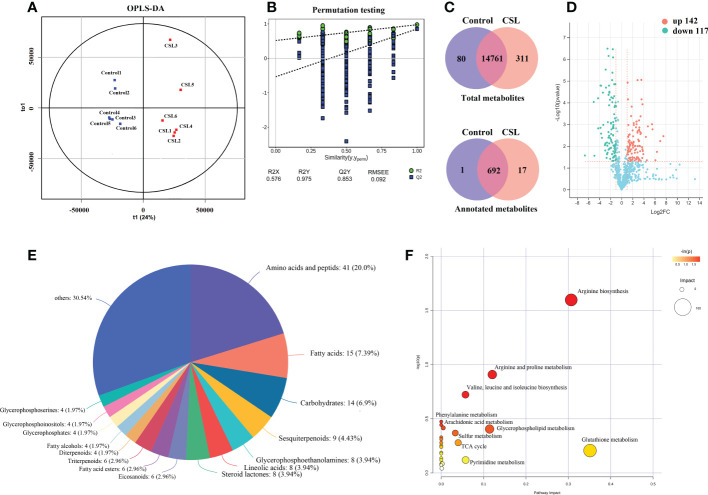
Overview of metabolomics in *Codonopsis pilosula*. OPLS-DA score plots **(A)**, OPLS-DA model permutation test **(B)**, Venn diagram **(C)**, volcano plot **(D)**, Classification of differential metabolites **(E)**, KEGG enrichment analysis of differential metabolites **(F)**.

The main differences in metabolite levels between the two groups were explored by OPLS-DA score plots (**Figure 3A**). The CSL group and the control group showed strong clustering, with no overlap. The permutation test of OPLS-DA model showed that R2X, R2Y (goodness-of-fit parameter) and Q2 (predictive ability parameter) were 0.576, 0.975, and 0.853, respectively, indicating the repeatability of the model (**Figure 3B**). Taken together, 17 of the 311 metabolites were annotated only in the CSL group (**Figure 3C**). The significantly differentially expressed metabolites (DEMs) were separated according to the criteria VIP > 1, p < 0.05 and |Log2(fold_change)| > 1 in the comparison between the CSL group and the control groups. There was a significant increase and decrease in the relative peak areas of 142 and 117 annotated metabolites in the CSL group, respectively (**Figure 3D** and **Supplementary Table S2**).

A total of 259 DEMs were categorised into 16 classes based on the HMDB database (**Figure 3E**). The metabolites were grouped into classes of amino acids and peptids (41 metabolites), fatty acids (15), carbohydrates (14), sesquiterpenoids (9), and others, such as steroids, lineolic acids and glycerophosphoethanolamines. In addition, we mapped DEMs to KEGG pathways, most of which were enriched in amino acid biosynthesis and metabolism, such as arginine, valine, leucine, isoleucine, phenylalanine, pyrimidine, glutathione, glycerophospholipids, and aromatic metabolisms (observed in **Figure 3F**).

### 3.4 Overview of phytometabolites

**Figure 4:**
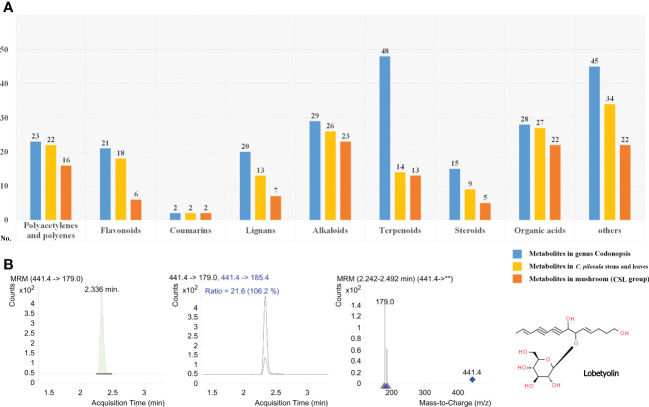
Phytometabolites identified in the fruiting body of *P. ostreatus.* **(A)** Annotation and classification of phytometabolites identified in CSL group; **(B)** Product ion mass spectra of [M+COOH]^-^ ion of lobetyolin and chromatography peak of a representative CSL sample (retention time 2.336 min).

More than 230 phytometabolites from *Codonopsis* have been described in previous studies (Gao et al., 2018). Currently, the metabolomics of *C. pilosula* have revealed a large number of phytometabolites, including polyacetylenes and polyenes, flavonoids, coumarins, lignans, alkaloids, terpenoids, steroids, organic acids, and other metabolites. In the present study, an abundance of phytometabolites were identified in *P. ostreatus* cultured on CSL (**Figure 4A** and **Supplementary Table S3**). In total, 16 polyacetylenes or polyenes, 6 flavonoids, 2 coumarins, 7 lignans, 23 alkaloids, 13 terpenoids, 5 steroids, 22 organic acids, and 22 other metabolites were detected from both of positive or negative mode. Differential phytometabolites were shown in **Table 2**.

**Table 2:** Differential pythometabolites for comparison bewteen mushrooms from CSL and control group.

Metab_ID	Apex_m/z	Mode	Adduct	Retention time	Log2(Fold_Change)	P_Value	Putative_Metabolite	Putative_Formula	Pubchem_ID
Polyacetylenes, polyenes and their glycosides									
metab_14533	558.231	neg	[M-H]-	1.1940	4.09	0.0451	Lobetyolinin or Codonopilodiynoside B or C or Tangshenyne B	C26H38O13	5459227
metab_1740	234.1256	pos	[M+H]+	1.9463	1.31	0.0049	lobetyol	C14H18O3	5807986
metab_556	252.1361	pos	[M+H]+	2.2045	1.29	0.0009	Pilosulyne A or G	C14H20O4	
metab_520	250.1205	pos	[M+H]+	3.2420	1.76	0.0257	Pilosulyne B	C14H18O4	
metab_2005	236.1412	pos	[M+H]+	2.8725	1.60	0.0029	Pilosulyne C or tetradeca-4E,8E,12E-triene-10-yne-1,6,7-triol	C14H20O3	
metab_481 metab_13480	254.1518	pos neg	[M+H]+ [M-H]-	2.8725 2.8440	1.12 3.06	0.0108 0.0203	Pilosulyne F	C14H22O4	
metab_7627	412.1733	neg	[M-H]-	2.6986	3.23	0.0570	Tangshenyne A	C20H28O9	
Flavonoids and their glycosides									
metab_9173	270.0528	neg	[M-H]-	3.6479	15.85	0.3318	apigenin	C15H10O5	5280443
metab_1164	300.0633	pos	[M+H]+	0.5140	-1.16	0.0003	chrysoeriol	C16H12O6	5280666
metab_6323	438.1162	pos	[M+H]+	0.6262	-14.94	0.0002	5,7,3’,5’-tetrahydroxy-flavone-O-glucopyranoside		
Coumarins									
metab_8610	186.0316	neg	[M-H]-	2.0358	5.92	0.0564	angelicin or psoralen	C11H6O3	10658 or 6199
Lignans and their glycosides									
metab_14820	372.1420	neg	[M-H]-	0.5991	1.97	0.3364	tangshenoside II or syringin	C17H24O9	5321613 or 5316860
metab_14115	364.1733	neg	[M-H]-	1.6797	1.66	0.0604	ethyl-syringin	C16H28O9	
metab_4792 metab_13197	360.1572	pos neg	[M+H]+ [M-H]-	4.6248 3.5978	2.36 1.91	0.1047 0.0885	lariciresinol	C20H24O6	332427
metab_8216	206.0579	neg	[M-H]-	1.3373	3.35	0.0059	lanceolune A	C11H10O4	56964234
metab_1994 metab_14385	220.0735	pos neg	[M+H]+ [M-H]-	2.8259 1.3817	-.1.19 -4.06	0.0005 0.0027	lanceolune B	C12H12O4	56964235
Alkaloids and their glycosides									
metab_9797	267.1471	neg	[M-H]-	6.7439	4.33	0.0716	codonopsine	C14H21NO4	442631
metab_12861	237.1365	neg	[M-H]-	4.7217	1.04	0.1079	codonopsinine	C13H19NO3	10889945
metab_6157 metab_8499	283.1420	pos neg	[M+H]+ [M-H]-	0.9620 1.8046	13.60 3.63	0.0860 0.0878	codonopsinol	C14H21NO5	44570118
metab_9124	253.1314	neg	[M-H]-	3.4969	1.40	0.0934	Codonopsinol B	C13H19NO4	44570115
metab_210	255.1107	pos	[M+H]+	0.6262	12.00	0.0169	radicamine A	C12H17NO5	10083681
metab_14024	187.0633	neg	[M-H]-	1.8205	6.86	0.0090	methoxy-4-formyl quinoline	C11H9NO2	219312
metab_8245	168.0687	neg	[M-H]-	1.3965	1.52	0.0644	norharman	C11H8N2	64961
metab_5167	160.0847	pos	[M+H]+	3.1191	2.49	0.0053	n-butyl allophanate	C6H12N2O3	5315565
Terpenoids and their glycosides									
metab_4566	232.1463	pos	[M+H]+	5.8597	5.06	0.0013	Atraetylenolide II	C15H20O2	
metab_2636	248.1412	pos	[M+H]+	6.8433	4.59	0.0025	Atraetylenolide III or atraetylenolide	C15H20O3	
metab_10411	468.3967	neg	[M-H]-	9.1038	-1.88	0.0059	taraxeryl acetate or β-amyrin acetate	C32H52O2	94225
metab_10523	456.3603	neg	[M-H]-	9.6316	-2.16	0.0156	oleanolic acid	C30H48O3	10494
metab_14048	140.0473	neg	[M-H]-	1.7885	1.95	0.0206	5-methoxymethyl-2-furaldehyde	C7H8O3	74711
Steroids and their glycosides									
metab_2780	408.3392	pos	[M+H]+	7.7208	-1.58	0.0258	Δ7-stigmasta-7-ene-3-one	C29H44O	
metab_10523	456.3603	neg	[M-H]-	9.6316	-2.16	0.0156	Δ5,22-stigmasteryl-β-D-glucoside	C30H48O3	
Organic acids and their glycosides									
metab_579 metab_4664	166.0993	pos pos	[M+H]+ [M+H]+	4.0939 5.2717	2.68 1.78	0.0230 0.0088	2,4-nonadlenic acid	C10H14O2	
metab_4882	328.2249	pos	[M+H]+	4.2457	1.87	0.0383	9,12,13-trihydroxy-10,15-octadecadienoic acid	C18H32O5	5312876
metab_5507	168.0422	pos	[M+H]+	2.1897	1.94	0.0098	vanillic acid	C8H8O4	8468
metab_1769	138.0316	pos	[M+H]+	2.0211	1.40	0.0094	4-hydroxy benzoic acid	C7H6O3	135
Others									
metab_7640	264.1572	neg	[M-H]-	2.7155	3.10	0.0059	n-hexyl-β-D-glucopyranoside	C12H24O6	
metab_6868 metab_13661	236.1259	neg neg	[M-H]- [M-H]-	1.2805 2.4970	1.34 1.56	0.0006 0.0035	n-butyl-β-D-fructofuranoside or n-butyl-α-D-fructofuranoside	C10H20O6	13386213
metab_13841 metab_8836	262.1416	neg neg	[M-H]- [M-H]-	2.1311 2.6046	1.13 1.87	0.0435 0.0000	(E)-2-hexenyl-β-D-glucopyranoside or (Z)-3-hexenyl-β-D-glucopyranoside	C12H22O6	5318046
metab_13408	276.1572	neg	[M-H]-	3.0260	3.75	0.0139	hexyl-α-D-fructofuranoside	C13H24O6	
metab_12734	426.2101	neg	[M-H]-	5.2579	-2.80	0.0480	hexyl-β-D-glucopyranosyl-β-D-glucopyranoside	C18H34O11	14655096
metab_8913	358.1263	neg	[M-H]-	2.7961	2.22	0.0447	sweroside	C16H22O9	161036
metab_4847	152.0473	pos	[M+H]+	4.3818	3.53	0.0440	vanillin	C8H8O3	1183
metab_6746	368.1107	neg	[M-H]-	0.5991	3.49	0.0373	3-O-Caffeoylquinic acid methyl ester	C17H20O9	6476139

Overall, different types of pythometabolites were obviously accumulated (|Log2(fold_change)| > 1) in the fruit bodies obtained from the CSL group. In brief, 8 polyacetylenes or polyenes (lobetyol, lobetyolinin, pilosulyne and etc.), 2 flavonoids or coumarins (apigenin and angelicin), 5 lignans (syringin, lariciresinol and lanceolune), 7 alkaloids (codonopsine, codonopsinol, radicamine A and etc.), 3 terpenoids (atraetylenolide, atraetylenolide and etc.), 4 organic acids (nonadlenic acid, vanillic acid and etc.), and 7 other metabolites (glucopyranoside, fructofuranoside, sweroside, vanillin and etc.) have been reported in previous reports. Pharmacological evidences confirmed that most of them have different effects on health.

### 3.5 Active ingredients

The presence of polyacetylenes and polyenes, an important group of active ingredients in the roots of *C. pilosula*, is detected in other tissues, such as roots, stems, leaves and flowers. As shown in **Table 2**, 8 DEMs in the comparison of the CSL group and the control group were identified as polyacetylenes, polyenes and their glycosides. Lobetyol (metab_1740) is a major ingredient of *C. pilosula* that has multiple pharmacological effects, including anti-inflammation, antiviral and proliferation inhibition functions (Shen et al., 2016). Similarly, lobetyolinin (metab_14533), the bis-glucosylated forms of the lobetyol, has been shown various bioactive properties such as anti-inflammation, anticancer, antioxidative, and xanthine oxidase inhibitiory properties (Bailly, 2021). However, lobetyolin, the mono-glucosylated forms of lobetyol, was not detected in the metabolomics. In addition, pilosulynes A-G (metab_556, metab_520, metab_2005, metab_481 and metab_13480) were firstly identified as five polyynes and two polyenes from *C. pilosula* roots. Among them, pilosulyne F has been reported to possess specific bioactive properties, such as anti-HCV (hepatitis C virus) activity (Liao et al., 2015).

Flavonoids, coumarins and their glycosides are widely distributed among medicinal plants. A large number of studies have confirmed that flavonoids and coumarins have many health benefits for the human diet. Here, 4 DEMs were identified in the metabolomics dataset. Evidences have demonstrated that apigenin (metab_9173) has a variety of pharmacological effects on diabetes, amnesia, Alzheimer’s disease, depression, insomnia and cancer (Salehi et al., 2019). In addition, a recent study supported the efficacy of angelicin or psoralen (metab_8610) isolated from medicinal plant psoraleae, including anti-inflammatory antibacterial effects, and osteogenesis effects, as well as immune modulation (Li et al., 2018).

Lignans are widely found in the different tissues of plant such as roots, stems, rhizomes, leaves, flowers, seeds, fruits, xylem, and resins. Previous studies have demonstrated that lignans and their derivatives possess various bioactive properties, including anticancer, antioxidant, antiviral, and anti-asthmatic activities (Xu et al., 2022). In our study, 7 DEMs of lignans were shown in **Table 2**. In recent years, some studies have suggested that both syringin (metab_14820) and ethyl-syringin (metab_14115) may enhance humoral and cell-mediated immunity in immune-deficient mice (Gao et al., 2019).

Alkaloids are one of the largest group of phytometabolites found in many medicinal herbs, and have wide applications in tthe reatment, health care, and positive prevention fields. The pharmacological mechanism of bioactive alkaloids has been the subject of much research in recent years. For example, total alkaloids from *C. pilosula* roots may promote differentiation of neurite PC12 cells by strengthening the upstream phase of the MAPK-dependent signalling pathway (Liu et al., 2003). In this study, 9 DEMs in CSL versus control group comparison were identified as alkaloids. Codonopsine (metab_9797), codonopsinine (metab_12861), and codonopsinol (metab_6157, metab_8499 and metab_9124), are pyrrolidine and polyhydroxy alkaloids and exhibit antimicrobial activity against MRSA (El-Nezhawy et al., 2019). Additionally, radicamine A (metab_210), a pyrrolidine alkaloid, could markedly inhibit the absorption of glucose in the small intestine and would be developed as a new drug for diabetes treatment (Liu et al., 2010).

Terpenoids are effective and active components of many medicinal plant. In recent decades, a large amount of terpenoids from plants or fungi have been used extensively in products such as food, drug, health supplements, and biofuels. Our analysis resulted in 5 DEMs annotated as terpenoids. Among them, atraetylenolide (metab_4566 and metab_2636) is the main active ingredient that has been isolated from *Atractylodes* rhizomes with anti-inflammatory, gastroprotective and neuroprotective effects (Zhou et al., 2021). Meanwhile, oleanolic acid (metab_10523) and its derivatives possess different pharmacological properties, including anticancer, anti-osteoporosis, antibacterial, antidiabetic, and haemolytic effects (Ayeleso et al., 2017; Gupta, 2022). However, oleanolic acid (metab_10523) was down-regulated in the CSL group compared to the control group.

Steroids, organic acids and other phytometabolites are also effective ingredients of medicinal herbs and possess broad pharmacological activities for human health. More recently, organic acid derivatives have also been applied in plant protection against pathogens and insect pests (Izquierdovega et al., 2020). Here, 5 DEMs were found to be organic acids. In detail, multiple pharmacological studies have demonstrated that vanillic acid (metab_5507) and vanillin (metab_4847), two phenolic acid in numerous edible plants, have beneficial health effects on inflammation and oxidative stress (Bezerra-Filho et al., 2019). In addition, sweroside (metab_8913), a secoiridoid glycoside, has been discussed previously in some detail, including different effects such as antibacterial, antioxidant, anticancer, hepatoprotective, gastroprotective, sedative and and neuroprotective activities (Ouyang and Xu, 2019; Zhang et al., 2019).

### 3.6 Detection of lobetyolin

In the Chinese Pharmacopeia 2020 edition, lobetyolin (mono-glucosylated forms of lobetyol) used as the standard composition to assess the quality of Radix Codonopsis. With respect to previous studies, it is more common to increase the sensitivity for the detection of lobetyolin by applying electrospray ionization (ESI) in negative mode. As shown in **Figures 4B**, lobetyolin was identified from the standard reference by analysis for ion fragmentation patterns. None of this components was detected in any of the control group samples. In contrast, the content of lobetyolin in the CSL group samples was 58.66 ± 15.27 mg/kg (0.0058%).

Our previous study reported similar findings (Zeng et al., 2022b). In the case of *C. pilosula*, the content of lobetyolin in the agro-waste residues (dried stems and leave) were 0.142 ± 0.011 mg/g (0.0142%). Despite that the general content in fresh leaves (0.039%) and stems (0.027%) of *C. pilosula* was not high, in earlier studies, lobetyolin in roots was found to have a similar or slightly higher content (0.01-0.07%) as a result of the different detection method (Chen et al., 2020). Overall, our result showed that *P. ostreatus* grown with CSL might absorb a certain amount of lobetyolin and have potential health care value in improving immunity.

## 4 Conclusion

In this study, we analysed and compared chemical constituents derived from *P. ostreatus* cultured with the agricultural waste materials from *C. pilosula*. Metabolites were classified and identified by our integrated bioinformatic pipeline Based on UPLC-MS data, a total of 710 metabolites were identified; 259 were identified as DEMs and grouped into the following categories: amoni acids and peptids, fatty acids, carbohydrates, steroids, flavonoids, and others. Metabolic profiling of *C. pilosula* tissues implied that polyacetylenes, polyenes, flavonoids, alkaloids, steroids, terpenoids, and organic acids were accumulated in the *P. ostreatus* grown with CSL. In addition, lobetyol (a major ingredient of *C. pilosula*) and lobetyolinin (the bis-glucosylated forms of the lobetyol) were also found in the metabolomics dataset of the CSL group samples, and lobetyolin (the mono-glucosylated forms of the lobetyol) was quantified accurately in the same samples by UPLC-MS/MS. In light of our findings, many active compounds from *C. pilosula* can be absorbed and accumulate in the fruiting bodies of *P. ostreatus*. Oyster mushroom cultured with the agricultural waste materials of *C. pilosula* (stems and leaves) could have very high health care value in nutritional therapy.

## Data availability statement

The original contributions presented in the study are included in the article/**Supplementary Material**. Further inquiries can be directed to the corresponding authors.

## Author contributions

XZ, JL, XC, and SG discussed and plan the work. XZ, JL and XL conducted the experiments. XZ, TC, JC carried out the data analysis, created the figures, and drafted the initial manuscript. XZ, JL XC and SG wrote the manuscript. All authors contributed to the article and approved the submitted version.

## Supplementary material

Supplementary Figure 1 The standard curve of lobetyolin.

Supplementary Table 1 Overview of metabolomics data.

Supplementary Table 2 Metabolites annotated with HMDB and KEGG databases.

Supplementary Table 3 Phytometabolites in *Codonopsis* from metabolomics data.
