# Supplementary figures and images for: Utilization of functional agro-waste residues for oyster mushroom production: Nutritions and active ingredients in healthcare
Source: Front Plant Sci. 2023 Jan 4;13:1085022. doi: 10.3389/fpls.2022.1085022 (PMC9846735; doi:10.3389/fpls.2022.1085022)

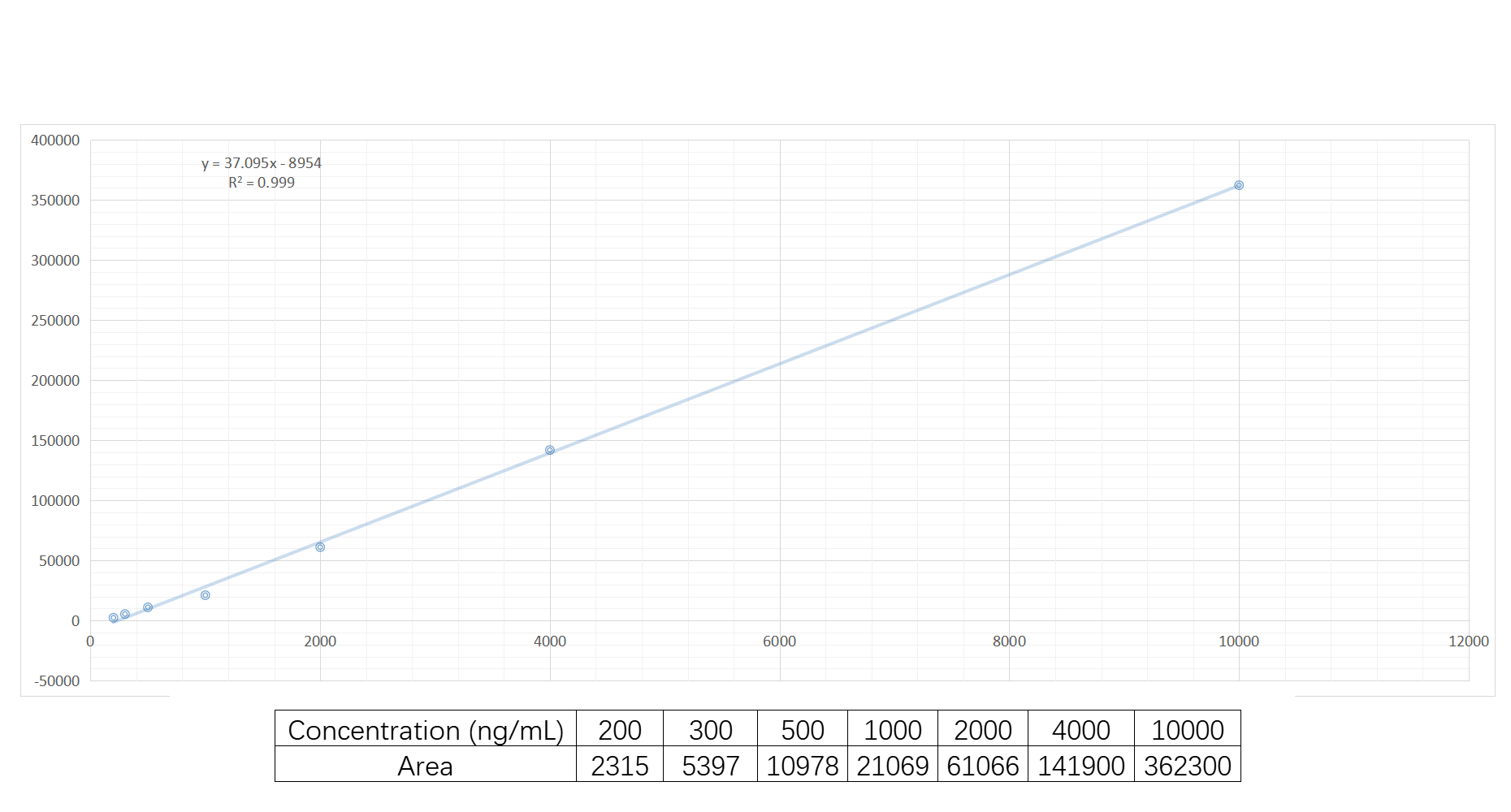

Supplement: Supplementary Figure 1 — The standard curve of lobetyolin. [file Image_1.tif]
